# Simultaneously Uncovering the Patterns of Brain Regions Involved in Different Story Reading Subprocesses

**DOI:** 10.1371/journal.pone.0112575

**Published:** 2014-11-26

**Authors:** Leila Wehbe, Brian Murphy, Partha Talukdar, Alona Fyshe, Aaditya Ramdas, Tom Mitchell

**Affiliations:** 1 Machine Learning Department, Carnegie Mellon University, Pittsburgh, Pennsylvania, United States; 2 Center for the Neural Basis of Cognition, Carnegie Mellon University, Pittsburgh, Pennsylvania, United States; 3 School of Electronics, Electrical Engineering and Computer Science, Queen's University, Belfast, United Kingdom; 4 Supercomputer Education and Research Centre, Indian Institute of Science, Bangalore, Karnataka, India; University of Leicester, United Kingdom

## Abstract

Story understanding involves many perceptual and cognitive subprocesses, from perceiving individual words, to parsing sentences, to understanding the relationships among the story characters. We present an integrated computational model of reading that incorporates these and additional subprocesses, simultaneously discovering their fMRI signatures. Our model predicts the fMRI activity associated with reading arbitrary text passages, well enough to distinguish which of two story segments is being read with 74% accuracy. This approach is the first to simultaneously track diverse reading subprocesses during complex story processing and predict the detailed neural representation of diverse story features, ranging from visual word properties to the mention of different story characters and different actions they perform. We construct brain representation maps that replicate many results from a wide range of classical studies that focus each on one aspect of language processing and offer new insights on which type of information is processed by different areas involved in language processing. Additionally, this approach is promising for studying individual differences: it can be used to create single subject maps that may potentially be used to measure reading comprehension and diagnose reading disorders.

## Introduction

Story understanding is a highly complex cognitive process that combines the low level perception of individual words, representing their meanings and parts of speech, understanding the grammar of sentences and their meanings, tying these sentence meanings together into a coherent understanding of the story plot and the evolving beliefs, desires, emotions, and actions of story characters. Story understanding and word and sentence processing have long been central topics of study across diverse fields including linguistics, computer science [1], cognitive science [2], literature and philosophy [3].

Due to this complexity, most experimental brain imaging studies of language processing have focused on just one aspect of language at a time, via carefully controlled experiments. For example, researchers have searched for brain regions where neural activity increases or decreases when the input stimulus is a word, in contrast to a non-word letter string [4], or a sentence with simple versus complex syntax [5], or a sentence with expected versus unexpected meaning [6]. These experiments require carefully controlled, hand-tailored textual stimuli that vary solely along one dimension of interest, raising the question of how much these findings reflect language processing in complex every-day use.

One of the main questions in the study of language processing in the brain is to understand the role of the multiple regions that are activated in response to reading. A network of multiple brain regions have been implicated in language [5], [7], and while the view of the field started with a simplistic dissociation between the roles of Broca's area and Wernicke's area, the current theories about language comprehension are more complex and most of them involve different streams of information that involve multiple regions (including Broca's and Wernicke's). Because of the complexity of language, the different experimental setups and the different hypotheses tested, different models have emerged leading to little agreement in the field, including on fundamental questions such as: Are language regions specific to language? [7]. There has been disagreement as well about other questions such as the role of the different "language" regions and the differentiation between regions processing syntax and regions processing semantics. [8] has found no regions to be responsive exclusively to syntactic or semantic information, while [9] has found regions in the Inferior Frontal Gyrus (IFG) that exclusively process syntax or semantics. Different models of meaning integration have also been proposed that disagree on the order in which semantic and syntactic information is accessed as a word is encountered, as well as on the order of integration of this information [10], [11].

We present in this paper a novel approach that can be used to answer these questions, as well as initial results that show that the different language processes are represented by different distributions of brain areas. Our data-driven approach studies the type of information being represented in different parts of the brain during a naturalistic task in which subjects read a chapter from *Harry Potter and the Sorcerer's Stone* [12]. We extract from the words of the chapter very diverse features and properties (such as semantic and syntactic properties, visual properties, discourse level features) and then examine which brain areas have activity that is modulated by the different types of features, leading us to distinguish between brain areas on the basis of which type of information they represent.

Our approach differs in multiple key respects from typical language studies. First, the subjects in our study read a non-artificial chapter, exposing them to the rich lexical and syntactic variety of an authentic text that evokes a natural distribution of the many neural processes involved in diverse, real-world language processing. Second, our analysis method differs significantly from studies that search for brain regions where the magnitude of neural activity increases along one stimulus dimension. Instead, our approach is to train a comprehensive generative model that simultaneously incorporates the effects of many different aspects of language processing. Given a text passage as input, this trained computational model outputs a time series of fMRI activity that it predicts will be observed when the subject reads that passage. The text passage input to the model is annotated with a set of 195 detailed features for each word, representing a wide range of language features: from the number of letters in the individual word, to its part of speech, to its role in the parse of its sentence, to a summary of the emotions and events involving different story characters. The model makes predictions of the fMRI activation for an arbitrary text passage, by capturing how this diverse set of information contributes to the neural activity, then combining these diverse neural encodings into a single prediction of brain-wide fMRI activity over time.

Our model not only accounts for the different levels of processing involved in story comprehension; it goes further by explicitly searching for the brain activity encodings for individual stimuli such as the mention of a specific story character, the use of a specific syntactic part-of-speech or the occurrence of a given semantic feature. The resulting trained model extrapolates from the training data to make testable predictions of the brain activity associated with novel text passages with may vary arbitrary in their content. In training this generative model we make minimal prior assumptions about the form of the hemodynamic response that relates neural activity to observed fMRI activity, instead allowing the training procedure to estimate the hemodynamic response separately for each distinct story feature at each distinct voxel; it has been shown that the hemodynamic response varies across different regions of the brain [13]. We also employ a novel approach for combining fMRI data from multiple human subjects, which is robust to small local anatomical variabilities among their brains. This approach allows us to produce more accurate population-wide brain representation maps by using data from multiple subjects, while avoiding the major problem associated with averaging voxel-level data across multiple subjects: the bias in favor of regions were subjects share the same smooth representation.

To validate this modeling technique, we show below that the predictions of our trained model are sufficiently accurate to distinguish which of two previously unseen short text passages is being read, given only the observed fMRI brain activity, with an accuracy of 74%. This accuracy is significantly higher than chance accuracy (50%), with 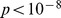. While the exact numerical value of the accuracy might not be particularly revealing, the fact that we can obtain such a statistically significant result is to our knowledge a novel discovery. It has not been shown previously that one could model in detail the rapidly varying dynamics of brain activity with fMRI while reading at a close to normal speed. This finding has important significance for the future study of reading and language processing, specifically given the new trend in cognitive neuroscience to shift away from experiments with artificial, controlled stimuli to using natural stimuli that mimic real life conditions [14] in order to obtain more generalizable conclusions.

Reporting accuracy of the trained model predictions is however not the main contribution of this paper. We also use the brain activity encodings of different story features learned by the trained model – including perceptual, syntactic, semantic, and discourse features – to provide new insights into where and how these different types of information are encoded by brain activity. We align and contrast these results with several previously published studies of syntax, semantics, and models of the mental states and social interactions with others. In this paper, we use the term "semantic features" to refer to the lexical semantic properties of the stimulus words, and use "discourse features" to refer to discourse semantics of the story.

The experiments in this paper use a particular set of 195 features, and provide a solid proof of concept of the approach. However, this approach is flexible and capable of capturing additional alternative hypotheses by changing the time series of features used to describe the sequence of words in the story. We plan to use this method in the future to test and contrast competing theories of reading and story understanding. As long as different theories can be characterized in terms of different time series of annotated story features, our approach can compare them by training on these alternative feature sets, then testing experimentally which theory offers a better prediction of brain data beyond the training set.

Our approach is analogous to earlier work that trained a computational model to predict fMRI neural representations of single noun meanings [15]. However, here we extend that approach from single nouns and single fMRI images, to passages of text in a story, and the corresponding time series of brain activity. This work is also analogous to recent work analyzing fMRI from human subjects watching a series of short videos where a large set of objects were identified and annotated with semantic features that were then mapped to brain locations [16], though that work was restricted to semantic features and did not include language stimuli. Our approach is the first to provide a generative, predictive model of the fMRI neural activity associated with language processing involved in comprehending written stories.

## Materials and Methods

### Data acquisition

FMRI data was acquired from 9 right-handed native english speakers (5 females and 4 males) aged 18–40 years, while they read chapter 9 of *Harry Potter and the Sorcerer's Stone* [12] (one subject's data was discarded due to artifacts we could not remove). All subjects had read the Harry Potter book series, or seen the movie series prior to participating in the experiment, and gave their written informed consent approved by the Carnegie Mellon University Institutional Review Board. All the subjects therefore were familiar with the characters and the events of the book, and were reminded of the events leading up to chapter 9 before the experiment. The chapter was read using Rapid Serial Visual Presentation (RSVP): the words of the chapter were presented one by one in the center of the screen, for 0.5 s each. The 5000 word chapter was presented in 45 minutes. Before the experiment, we asked the subjects to get used to reading in RSVP by providing them with a practice video of an unrelated text. The word presentation rate was deemed comfortable by the subjects. More details are presented in Appendix A of File S1.

### fMRI procedure

Functional images were acquired on a Siemens Verio 3.0T scanner at the Scientific Imaging & Brain Imaging Center at Carnegie Mellon University, using a T2* sensitive echo planar imaging pulse sequence with repetition time (TR)  = 2 s, echo time  = 29ms, flip angle  = 79°, 36 slices and 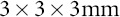 voxels.

### Computational model

We trained a computational model to predict the observed sequence of fMRI brain activity while the subjects read chapter 9 of *Harry Potter and the Sorcerer's Stone* [12]. To characterize the input time series of text (of which each word was shown for 0.5 s), a vector time series was created with 195 story features whose values change every 0.5 s. As illustrated in Fig. 2(a), these story features include syntactic features (the part of speech of every word, its ordinal position and dependency role in the sentence it belongs to), semantic features of individual words (derived from word dependency and document co-occurrence frequencies in large online corpora and then compressed using Non-Negative Sparse Embedding [17]), and low level percepts such as the number of letters in the current word. The list also includes a diverse set of discourse features. Because of how chapter 9 is structured, we decided to capture the narrative structure by using features that identify the different story characters and features that characterize the events they participate in: physical motions they perform, non-motion actions they perform and the emotions they experience. This chapter also contains frequent instances of directly quoted speech, and therefore we used the presence of dialog as a feature. Other narrative elements such as location did not vary or occur frequently in the chapter and therefore we excluded them. The set of 195 story features is described in Appendix B of File S1, which also contains the complete list of all the features (table 1) and examples of the feature values for given passages (table 2).

Because one fMRI image is acquired every 2 s, the model collapses the 0.5 s time series of story feature vectors by summing the story feature vectors associated with the four consecutive words presented in each 2 s interval. The result is a story features time series with values every 2 s, aligned to the timing of the fMRI data acquisition.

**Figure 1:**
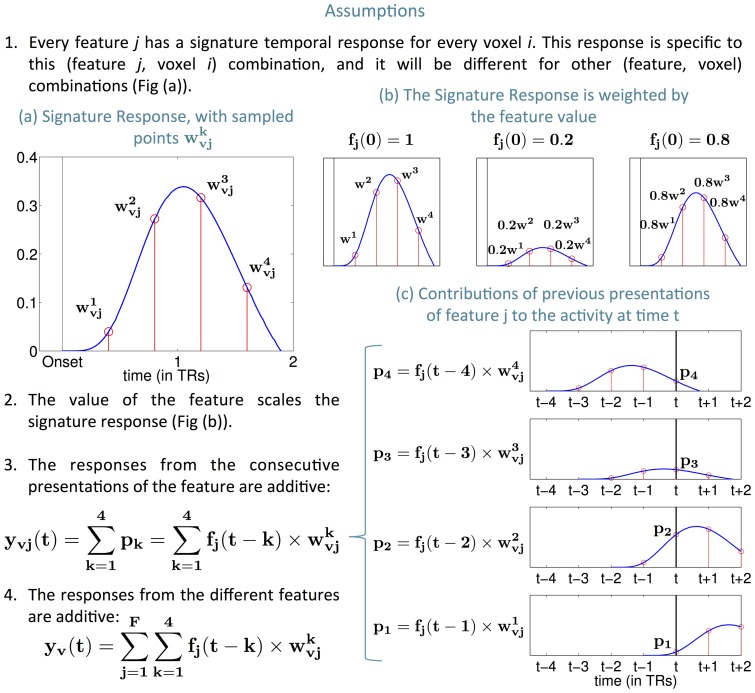
Time model of a voxel's response to the consecutive occurrences of the features of a story. Because of the hemodynamic response latency, the occurrence of a feature at time t will affect the activity of the voxel for several TRs after time t. This latency is accounted for by considering occurrences of features at previous TRs when modeling a voxel's activity at time t. (One TR is the repetition time needed to acquire one fMRI image, here we use a TR of 2 s).

The model predicts the neural activity at each voxel independently. It assumes that each time a particular story feature 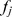 occurs, it will generate the same response signature in voxel 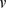, weighted by that feature's value. Since changes in the fMRI signal persist for approximately 8 s after neural activity and the signal is sampled with a period of 2 s, the model estimates this response signature for feature 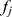 as a series of points 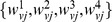 corresponding respectively to times 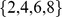 seconds after feature onset. The signal in a voxel 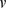 at time 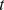 is therefore modeled as:
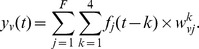



The activity at voxel 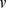 is the sum of the contributions of the 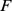 story features. Each feature 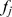
's contribution is the convolution of its magnitude over time with its temporal response signature at voxel 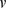. This is illustrated in Fig. 1 and more details are listed in Appendix C of File S1.

**Figure 2:**
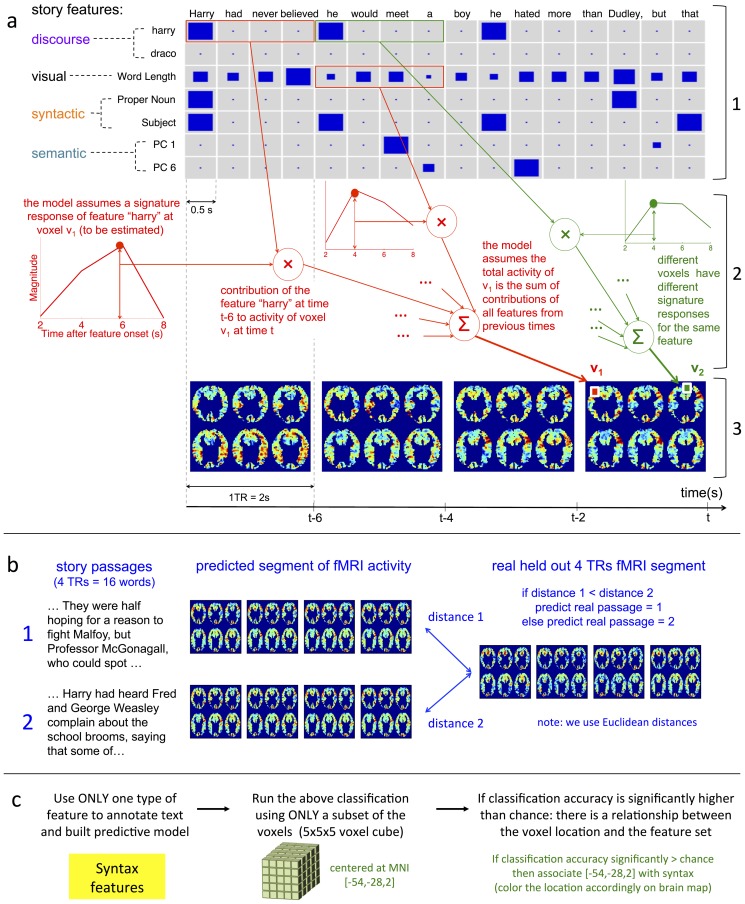
Illustration of the model and the classification task. **a**- (1) Diagram showing 7 of the 195 story features used to annotate a typical story passage. The size of each square indicates the magnitude of the feature. (2) Diagram of our generative model. The model assumes that the fMRI neural activity at each voxel at time 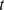 depends potentially on the values of every story feature for every word read during the preceding 8 s. Parameters learned during training determine which features actually exert which influence on which voxels' activity at which times. Response signatures shown here are hypothetical. A rectangle around 4 consecutive feature values indicates these values correspond to one time point and their magnitudes were summed. (3) Time course of fMRI volumes acquired from one subject while they read this specific story passage. Only 6 slices are shown per volume. **b**- Classification task. We test the predictive model by its ability to determine which of two candidate story passages is being read, given a time series of real fMRI activity held out during training. The trained model first predicts the fMRI time series segments for both of the candidate story passages. Then it selects the candidate story passage whose predicted time series is most similar (in Euclidean distance) to the held out real fMRI time series. The model's accuracy on this binary task is 74%, which is significantly higher than chance performance (50%), with 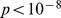. **c**- Diagram illustrating the approach to discover what type of information is processed by different regions. We choose one feature set at a time to annotate the text, and we run the entire classification task using only a subset of voxels centered around one location. If classification is significantly higher than chance, we establish a relationship between the feature set and the voxel location. We repeat for every feature set and every location and we use these relationships to build representation maps.

Every voxel's activity at time 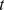 is thus a linear combination of all story features at the four preceding time points, where the specific linear combination is determined by the set of learned 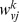 parameters. 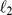-regularized linear regression was used to learn the very large set of parameters (Appendix D of File S1). The model is trained independently for each subject in the study. Note the parameters 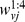 that represent a single time signature response are learned with no assumption on the shape of the response function, observed in fMRI time series. On average, we obtain for some types of features concave time series shapes that resemble the characteristic shape of the typical fMRI hemodynamic response (Appendix E of File S1). However, our model also allows for the possibility that certain story features evoke very complex time series of neural activity whose fMRI signatures vary greatly from the standard hemodynamic response to a single isolated impulse of neural activity. Consequently, for some types of features, we learn more complex impulse responses. We have tried using more time points to estimate the response (5 and 6 instead of 4), however we did not find any region in which the model improved significantly in performance (Appendix F of File S1). Because we already have a large number of covariates (195 features 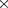 number of time windows) and a fixed number of samples, we chose to use 4 time points. Fig. 2(a) shows a summary of the predictive model.

### Whole Brain Classification

To evaluate the model's accuracy, a cross-validation approach was used in which the model was repeatedly trained and tested. In each cross-validation fold, only 90% of the story time series and associated fMRI data were used for training the model, while the remaining 10% were held-out as test data. We divided the held-out story times series and the associated fMRI data into non-overlapping time-series segments of length 20 TRs. Fig. 2(b) summarizes how the accuracy of model predictions was assessed (in that figure, the segments are of length 4TRs for simplicity but the concept is the same). We go through the held out 20 TRs fMRI time series; for each one of the time-series, we perform a classification task that aims to identify the correct 20 TR story passage out of two possible choices (the corresponding 20 TRs passage and another one chosen at random). The classification is done in two steps. (1) The model predicts the fMRI time series for each of these two passages, for each of the human subjects in the study (recall that a different model is trained for each human subject). The predicted fMRI time series for all 8 subjects are then concatenated to form a predicted *group fMRI time series* covering all subjects in the study. (2) The held out group fMRI time series (which also corresponds to the concatenation of the 8 subjects' time-series) is then compared to the two predicted group time series and the model is required to determine which of the two passages was being read when the observed group fMRI data was collected. To answer this two-choice classification task, the model chooses the passage whose predicted group fMRI time series is closest (in Euclidean distance) to the observed group fMRI time series.

Note that the chance-level performance in this two-way classification of text passages over the held-out data is 50%. Also note that both the learning and classification steps were done without averaging data over subjects or making assumptions on their brain alignment. Further details are provided in Appendix F of File S1. Finally, note that we repeat the classification of each fMRI segment a large number of times with different alternative choices to minimize the variance of the results. The boundaries of the passages we choose are arbitrary since the selection is made automatically and all of the story passages are constrained to be of the same size, i.e. the two test passages do not correspond to defined paragraphs or sections of the text. Because we pair each true passage with many other passages in different classification tasks and average the accuracy over all the tasks, we minimize confounds that might occur because two specific passages are extremely different in some way that is tangent to the information content we are studying.

### Uncovering Different Patterns of Representation

We wished to explore which story features mapped to which locations in the brain. To find this mapping the above classification approach was followed, but using **only one type of story feature at a time** to annotate the text passage (e.g. only the semantic features). Fig. 2(c) describes this approach. We also limited the predictions to a **small subset of the voxels** in a Searchlight-like [18] manner that we call concatenated Searchlight. This concatenated Searchlight uses a 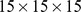
mm cube centered at one voxel location (corresponding to 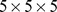 voxels). After normalizing the subjects to the MNI (Montreal Neurological Institute) space, we include in each cube the set of voxels from all subjects whose coordinates fall into the cube (subjects may differ in how many voxels they contribute to a particular cube because of the disparity in the size of their ventricles or the shape of the surface of their brain).

Our concatenated Searchlight is not equivalent to spatial or cross-participant smoothing because, again, the voxels associated with each subject are treated independently. The difference is discussed in Appendix H of File S1. Because the voxel cube used is larger than one voxel (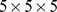 voxels), this method searches for regions with high accuracy across subjects while allowing for small anatomical variations among their brains.

By successively testing every **type of feature** 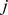 at every cube **location** 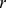, we determine in which brain regions each type of feature yields high classification accuracy. Our assumption is that, if using feature set 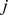 in location 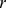 yields a high classification accuracy, then the activity in region 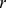 is modulated by feature set 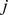, i.e. region 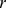 represents feature 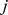. For example, if using part of speech features allows us to classify very accurately a region in the temporal pole, then this suggests that this region of the temporal pole is representing part of speech information.

To assess the significance of the classification accuracies an empirical distribution of chance level performance was estimated. We then corrected for multiple comparisons (Appendix F of File S1). From the classification results, we therefore obtain accuracy maps that allow us to determine where each type of information is represented by fMRI activity.

## Results and Discussion

### Whole Brain Classification Results

We compute the average classification accuracy of our model when predicting fMRI time series associated with text passages that were not observed during training. The model is able to classify which of two novel passages of the story is being read with an accuracy of 74%. This is significantly higher than chance accuracy, which is 50% in this balanced task (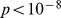), indicating that the model can indeed distinguish between the literary content of two novel text passages based on neural activity while these passages are being read.

The successful classification results we obtain indicate that, despite the low temporal resolution, it is possible to investigate the fast dynamic process of reading at a close-to-normal pace using fMRI, and to train a computational model of story comprehension that can successfully predict the time series of neural fMRI activity generated by reading novel passages of text. This model tracks multiple levels of processing of the story and links them to different brain areas. Our approach combines data from multiple subjects while allowing for subject-to-subject anatomical variability, makes minimal assumptions about the shape of the time series response to different story features in different brain regions, and learns the shape of these responses from observed data. As an extra advantage, authentic stories provide engaging experimental stimuli which helps subjects to remain alert.

We set out next to investigate how the different types of cognitive processes that underlie story reading are represented in the brain. For that purpose we ran the concatenated Searchlight approach, described in the methods section, using different input features and we constructed representation maps, which we discuss next.

### Different Patterns of Representation

**Figure 3:**
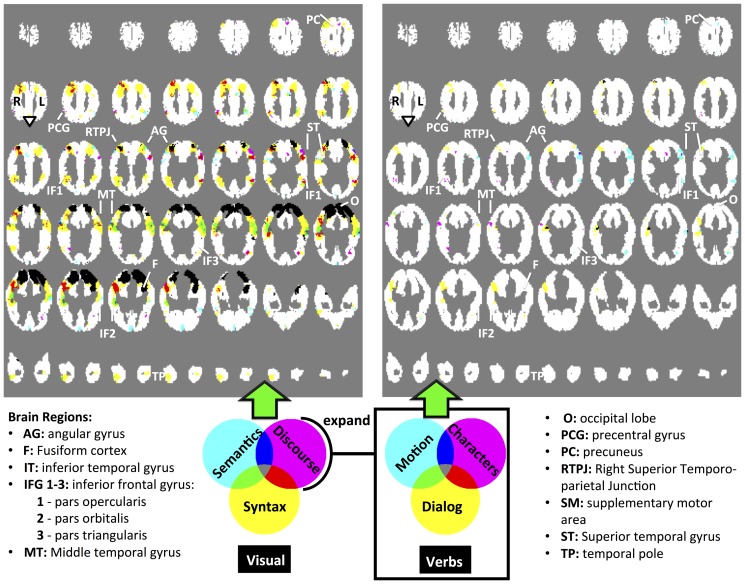
Accuracy maps revealing different patterns of representation of different reading processes. (**Left**) Voxels with significantly higher than chance classification accuracy when using different types of story elements as features, shown in different colors corresponding to the type of story elements. The brain used here is a superset of the brain of the 8 subjects, i.e. the union of all the voxel locations in the 8 brains. The slices are drawn such that they increase in the Z MNI-coordinate when going right to left, then bottom-up. Within each slice, the top of the slice corresponds to the posterior of the brain, and the right side of the slice corresponds to the left side of the brain. Each voxel location represents the classification done using a cube of 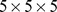 voxel coordinates, centered at that location, such that the union of voxels from all subjects whose coordinates are in that cube are used. (**Right**) Voxels with significantly higher than chance classification accuracy when using different types of discourse elements as features, shown in different colors corresponding to the type of discourse elements.

**Figure 4:**
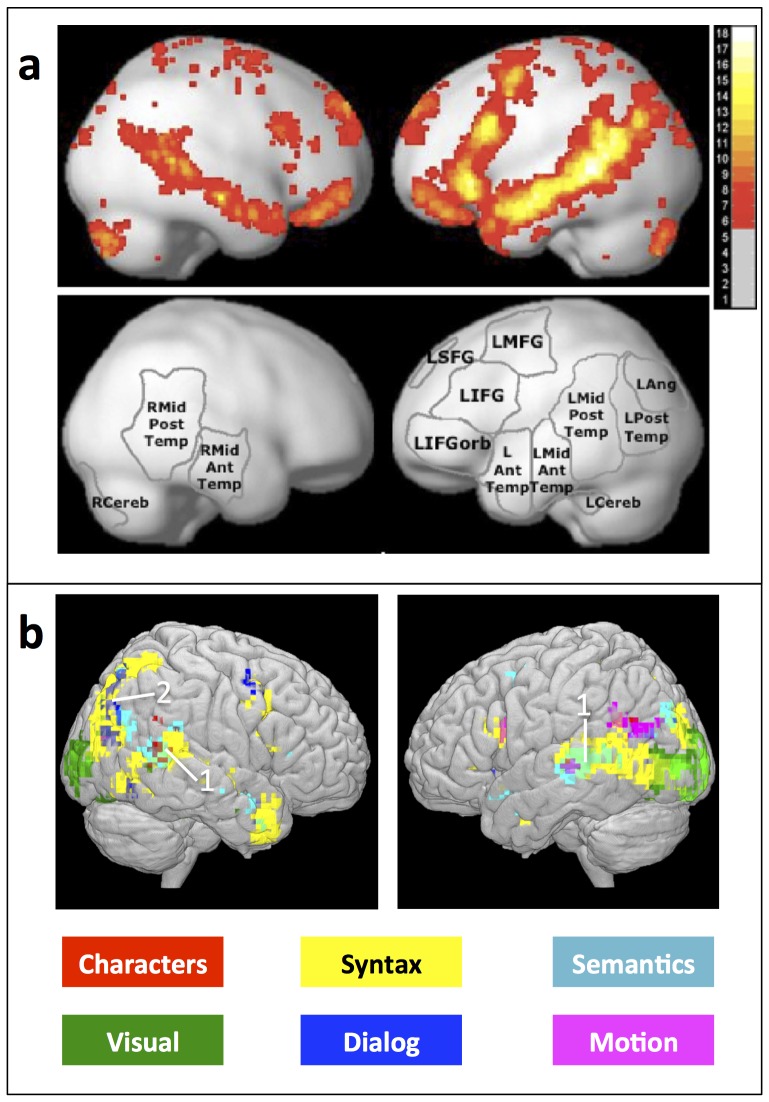
Map of the patterns of representation compared with the regions involved in sentence processing: our method recovers similar regions and differentiates them according to which information process they represent. **a**- Adapted from [8]: Top: A recently published probabilistic overlap map showing where sentence reading generates greater neural activity than perceiving nonword letter strings. The value in each voxel indicates how many of the 25 individual subjects show a significant (at 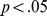, FDR-corrected) effect for the Sentences
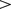
Nonwords contrast. Bottom: The main functional parcels derived from the probabilistic overlap map using an image parcellation (watershed) algorithm, as described in more detail in [7]. **b**- Results obtained by our generative model, showing where semantic, discourse, and syntax information is encoded by neural activity. Note this model identifies not just where language processing generates neural activity, but also what types of information are encoded by that activity. Each voxel location represents the classification done using a cube of 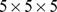 voxel coordinates, centered at that location, such that the union of voxels from all subjects whose coordinates are in that cube are used. Voxel locations are colored according to the feature set that can be used to yield significantly higher than chance accuracy. Light green regions, marked with (1), are regions in which using either semantic or syntactic features leads to high accuracy. Dark gray regions, marked with (2), are regions in which using either dialog or syntactic features leads to high accuracy.

Fig. 3(Left) shows the map of statistically significant classification accuracy (controlled at a false discovery rate of 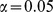) for the four categories of story features: semantics, syntactic, discourse features and visual features. Fig. 3(Right) offers a closer look at the different categories of discourse features. Fig. 4 (b) shows the learned map on the surface of a brain template. We did not find regions with significantly higher than chance accuracy along the medial wall and therefore we don't show it in Fig. 4(b). We discuss the different regions in this section.

#### Word Length

We find that the regions from which we can decode using the word length properties are in the occipital cortex, spanning the visual cortex (V1-4, VO1-2). This result is highly expected, and serves as an initial sanity check since the regions with high classification accuracy are mainly in the visual cortex. The visual regions are larger in the left hemisphere, spreading to the left fusiform cortex. This is most probably due to the activity of the Visual Word Form Area [19] that is being modulated by word length.

#### Syntax and Structure

Our results indicate that multiple areas in the brain represent language structure and syntax. Some of these regions are expected while others are somewhat surprising. Our syntax and structure features were composed of features related to part of speech and punctuation, grammatical role of a word in a sentence and the ordinal number of the word in the sentence. These features therefore capture a rich array of information: they are not only a measure of syntactic complexity but they also capture the different grammatical structures of the sentences in the text.

Fig. 8 in Appendix G of File S1 shows the breakdown of the syntax regions along our three types of features. In [20] the authors identified a network of regions where neural activity was correlated with the length of linguistic constituents. Using the sentence length feature, we were able to recover only the left temporo-parietal region that is reported (when using non-smoothed data - see Appendix G - we are also able to recover the left posterior superior temporal sulcus region that is reported). Interestingly, we find many more regions in the right temporo-parietal cortex that are related to sentence length. These regions are also modulated by the other syntactic features as well as by the presence of dialog. This indicates that these regions are modulated by the complexity and length of sentences. The right parietotemporal cortex has been implicated previously in verbal working memory processes [21] and has been shown to be more activated for good readers than for poor readers [22].

The strong right temporal representation of syntax that we found was not expected. Indeed we did not find other papers that report the large right hemisphere representation of sentence structure or syntax that we obtain. One reason might be that our syntax features are unique: whereas most experiments have approximated syntactic information in terms of processing load (length of constituents, hard vs easy phrase structure etc.) we model syntax and structure using a much more detailed set of features. Specifically, our model learns distinct neural encodings for each of 46 detailed syntax features including individual parts of speech, (adjectives, determiners, nouns, etc.) specific substructures in dependency parses (noun modifiers, verb subjects, etc.), and punctuation. Earlier studies considering only increases or decreases in activity due to single contrasts in syntactic properties could not detect detailed neural encodings of this type. We hypothesize that these regions have been previously overlooked.

The regions we find in the bilateral temporal cortices are related to both dependency role and part of speech features, indicating that they might be involved in both integration of the successive words and the representation of the incoming words. regions that are slightly more posterior represent part of speech features (features of the incoming words) and the ones that are slightly more anterior represent dependency roles (i.e. are implicated in word integration and sentence structure building). Regions in the bilateral temporal poles and the right IFG are representing dependency roles, indicating more high level processing, while the left IFG represents both dependency roles and parts of speech.

#### Lexical Semantics

Our model also found parts of the brain that represent semantics of individual words. Some of these areas such as the left superior and middle temporal gyrii and the left IFG have frequently been reported by others to represent semantics during language processing [23]. We found a right middle temporal representation of semantics. This is consistent with a theory of coarse semantic representation in the right hemisphere [2]. We also found semantic representation in the medial frontal cortex as well as the bilateral angular gyrii and the left pre-central gyrus.

#### Dissociation of Syntax and Semantics

The question whether the semantics and syntactic properties are represented in different location has been partially answered by our results. There seems to be a large overlap in the areas in which both syntax and semantics are represented. This is partially in alignment with what [8] found. The authors found that all the regions responsive to language stimulus were responsive to both syntax and semantics information. They were however able to distinguish between pure semantic information (word lists) and pure syntactic information (Jaberwocky) in some of the regions, leading them to conclude that in some of the regions syntactic and semantic information were not very closely represented and could be distinguished by voxel activity. They also found the lexical semantic information to be more strongly represented than the syntactic information. Using our natural story reading paradigm, we have found partially similar results: many regions in the bilateral temporal cortices seem to be coding both semantic and syntactic meaning, leading to one of two conclusions: either these brain regions process a meaning that is common to semantic and syntactic properties of words that are closely linked together, or our features are themselves representing information at the intersection of semantics and syntax that is related to the activity in that region. Furthermore, we find (1) regions that are selectively processing syntax and semantics and (2) that syntactic information is more widely and strongly represented. The difference could be due to the richness of our syntactic features and the additional fact that they indirectly measure verbal working memory and effort, which would recruit general purpose areas that exceed the language network.

#### Discourse and narrative features

Our results reveal a variety of brain regions that encode different information about story characters. Physical motions of story characters were represented in the posterior temporal cortex/angular gyrus, a region implicated in the perception of biological motion [24]. It has been shown that imagined biological motion also activates this area [24]. Processing the motions of the characters also modulated the activity of a region in the superior temporal sulcus, as well as in the left inferior frontal gyrus.

Presence of dialog among story characters was found to modulate activity in many regions in the bilateral temporal and inferior frontal cortices; one plausible hypothesis is that dialog requires additional processing in the language regions. More interestingly, it seems like presence of dialog activates the right temporo-parietal junction, a key theory of mind region [25]. This observation raises an exciting hypothesis to pursue: that the presence of dialog increases the demands for perspective interpretation and recruits theory of mind regions.

The identities of different story characters can be distinguished based on neural activity in the right posterior superior/middle temporal region. In [2] a "protagonist's perspective interpreter network" is outlined, based on a review of multiple studies. It encompasses among others the right posterior superior temporal gyrus. This region is also a classical theory of mind area [25], and has been found to encode facial identity [26].

### Differentiation of areas and stability of results

We therefore find a different representation for each type of features, with somewhat little specificity of the individual language regions. We suspect that these results, while revealing if considered at a coarse spatial scale, are however dependent on the analysis approach when the exact voxel locations are desired. To illustrate this point, we show in Fig. 9 and 10 in Appendix G of File S1 the results from running the same model as ours, with the change that the data was not smoothed spatially beforehand. There is a large variation in the boundaries of the regions, while the main general locations have some consistency.

The reason for the difference in the results is that our classification method relies on ridge regression and learns a different penalty parameter for each voxel. This leads to learning very high penalty parameters for noisy voxels, and very small ones for good voxels, effectively resulting in an automatic voxel selection (Appendix D of File S1). When the data is spatially smoothed, this disturbs the voxel selection, reduces the selection effect and brings down accuracy slightly, resulting in a smoother thresholding and more interpretable map such as the one in Fig. 4 and 8. It is however not straightforward to decide which method leads to more accurate spatial localization results. This observation really reveals the fickleness of brain imaging results, which are a general problem in the field, and their high dependence on even the analysis methods, which lead to different conclusions, especially when dealing with questions like specificity of regions. Analysis methods vary considerably between experiments, and it's not always clear which approach is more appropriate since multiple approaches can be statistically sound. This points to the urgency for establishing better standards and better methods that would be robust to such changes. We are currently working towards this goal.

An additional concern when looking at the regions identified for different features is that significance thresholding doesn't take into account that these different types of features have different statistical properties that influence their performance, and comparing them on the same metric introduces some arbitrariness. We discuss these issues in Appendix G of File S1, tables 3 and 4, and we show in Fig. 11 a map in which we color the top 1000 voxels per feature in terms of accuracy, instead of coloring the voxels that exceed the significance threshold.

### A comprehensive study of language processing

We have used our model to shed new light on what information is encoded by neural activity in different regions of the brain during story comprehension. Whereas previous research has shown which brain regions exhibit *increased brain activity* associated with different aspects of language processing, our results reveal in addition which brain regions *encode specific information* such as the identity of specific story characters. In recent research [8], a network of regions involved in language processing is obtained. It includes regions from the left angular gyrus to the left temporal pole, multiple left IFG regions, and multiple right temporal regions. That network is show in Fig. 4(a). Our own analysis, shown in Fig. 4(b), largely agrees with these findings, in terms of which regions exhibit language-related activity. However, as shown in Fig. 4(b), our analysis also reveals which of that neural activity is modulated by (and may therefore encode) specific perceptual, syntactic, semantic and discourse story features. Whereas previous work has studied some of these correspondences in isolation, the results presented here are the first to examine neural encodings of diverse story information at such a scale and across the brain in a realistic, story reading setting.

As illustrated in the above discussion, the model of reading introduced here can be used to study many aspects of reading simultaneously, without needing to vary just one dimension of the experimental stimulus at a time. This departure from the classical experimental setting has many advantages. We can use natural texts as stimuli, and study close-to-normal reading with its natural diversity of language constructs and attendant neural subprocesses. This model is also very flexible – given a rich enough stimulus, one can add additional stimulus features that one wishes to study. As suggested by [27], one could analyze an experiment with a new set of features without needing to collect new brain image data for each phenomenon of interest.

The rise of brain image decoding has already made the neuroimaging field aware of the difference between (a) approaches that use the presence/absence of a stimulus and (b) approaches that use the presence of different instantiations of the stimulus. For example [28] distinguishes between regions that identify the presence of faces and regions that process the characteristics of faces. Out of the regions that are modulated by the presence of a face, the authors determine which regions can be used by a classifier to decode which face was being seen. Using different instantiations of a stimulus (e.g. of a face) therefore allows us to find regions which encode the properties of the stimulus in consideration. In our experiment, we take this approach to the next level: there is only one stimulus (text) that is always being presented, and it is instantiated with a very large diversity (variations along a large number of dimension). More work is needed to understand more deeply how the different approaches of studying language tie together; and to understand how to combine what we can learn from experiments that rely on modeling the features of the stimuli (such as ours) versus experiments that contrast different types of information load (for example comparing stories to scrambled sentences and scrambled words such as in [29]). A compelling question that we have yet to answer is how much can we rely on modeling experiments, and how much can we stray from using controlled experiments. The similarity between our results and the literature we cited, and the fact that we reproduced many of these results using one modeling experiment only, are an encouraging first answer.

Furthermore, under the uncontrolled setting of our experiment, more work is needed in order to discount the effect of the correlation between the features sets. We obtain many regions which are related to multiple types of features, and it is crucial for our modeling approach to determine which of these associations are only due to the correlations between the feature sets. We are currently working on this problem and on expanding the computational methods we described here to give a clearer picture of the relationship between types of features and brain regions.

While the above discussion focuses on a map of group-wide language processing obtained from multiple subjects, it is also possible to use this approach to produce subject-specific reading maps. We suggest that our approach may be useful in the future to investigate language processing in a way that was not possible before. For example, one might test a hypothesis about how aphasic patients develop alternative processing routes by discovering the information encoded in each region for participants with aphasia and comparing the resulting distributions to controls. Similarly, subject-specific reading maps might be used to understand the cause of an individual's reading difficulties, and to better understand individual differences in reading processes. A further potential use is for pre-surgical mapping: this approach might help to identify, in parallel and with great precision, the patient-specific network of regions involved in language processing.

## Supporting Information

File S1 **List of appendices A-H including the detailed experimental procedures, textual annotations, description of the predictive model and classification setup, along with additional results.** Accompanying Website: http://www.cs.cmu.edu/afs/cs/project/theo-73/www/plosone/providing the fMRI data and feature annotations.
(PDF)
