# Correction: Simultaneously Uncovering the Patterns of Brain Regions Involved in Different Story Reading Subprocesses

**DOI:** 10.1371/journal.pone.0123148

**Published:** 2015-03-26

**Authors:** The PLOS ONE Staff

There is an error in the Data Availability statement. All data are not included in Appendix A. The correct statement is: The authors confirm that all data underlying the findings are fully available without restriction. All data are provided at http://datadryad.org/resource/doi:10.5061/dryad.gt413 and on the supplementary website: http://www.cs.cmu.edu/~fmri/plosone/<http://www.cs.cmu.edu/~fmri/plosone/

There is an error in the legend for S1 file. Please see the corrected S1 file legend below.

## Supporting Information

S1 File List of appendices A-H including the detailed experimental procedures, textual annotations, description of the predictive model and classification setup, along with additional results. (PDF)

## References

[1] Wehbe L, Murphy B, Talukdar P, Fyshe A, Ramdas A, Mitchell T. (2014) Simultaneously Uncovering the Patterns of Brain Regions Involved in Different Story Reading Subprocesses. PLoS ONE 9(11): e112575 doi:10.1371/journal.pone.0112575 25426840 10.1371/journal.pone.0112575 PMC4245107

