# Supplementary material for: Simultaneously Uncovering the Patterns of Brain Regions Involved in Different Story Reading Subprocesses
Source: PLoS One. 2014 Nov 26;9(11):e112575. doi: 10.1371/journal.pone.0112575 (PMC4245107; doi:10.1371/journal.pone.0112575)
Supplement: File S1 — List of appendices A-H including the detailed experimental procedures, textual annotations, description of the predictive model and classification setup, along with additional results. Accompanying Website: http://www.cs.cmu.edu/afs/cs/project/theo-73/www/plosone/providing the fMRI data and feature annotations. (PDF) [file pone.0112575.s001.pdf]

Appendix A. Experimental design

Material

Participants read chapter 9 of *Harry Potter and the Sorcerer's Stone* [Rowling, 2012]. We chose this chapter because it involves many characters and spans multiple locations and scenes. We chose a famous book series because we hypothesized all subjects already had characteristic mental representations of the different characters and locations, and that at least a part of this representation would remain constant throughout the reading of chapter 9. This assumption allows us to use data from the entire chapter to look for the representation of the different characters, e.g. the protagonist Harry Potter. In contrast, had we chosen an unfamiliar story in which we learn about the protagonist's personality throughout the text, the mental representation of this protagonist will arguably change more than Harry's would.

Participants

fMRI data was collected from 9 subjects (5 females and 4 males) recruited through Carnegie Mellon University, aged 18 to 40 years. The participants were all native English speakers and right handed. They were chosen to be familiar with the material: we made sure they had read the Harry Potter books or seen the movie series and were familiar with the characters and the story. All the participants were screened for safety, signed the consent form and were compensated for their participation. Data from one of the subjects was excluded from the analysis because of an artifact that was not removed by our preprocessing procedure.

Design

The words of the story were presented in rapid serial visual format [Buchweitz et al., 2009]. Words were presented one by one at the center of the screen for 0.5 seconds each (see Fig. 5). The background was gray and the font was black. We used MATLAB and the Psychophysics Toolbox extensions [Brainard, 1997, Pelli, 1997, Kleiner et al., 2007].

The chapter was divided into four runs, of approximately 11 minutes each. Subjects had short breaks between runs. Each run started with a fixation period of 20 seconds in which the subjects stared at a cross in the middle of the screen. The words presentation started after the fixation period. The total length of the runs was 45 minutes, during which about 5200 words were presented. Chapter 9 was presented in its entirety without modifications and each subject read the chapter only once.

Before the experiment, we supplied the subjects with a summary of the events preceding chapter 9 and a summary of the main characters and concepts in *Harry Potter and the Sorcerer's Stone* to refresh their memory. We also instructed them to practice rapid serial presentation by viewing a video that replicated the parameters of our design, but with another story (*The Tale of Peter Rabbit* [Potter, 2006]). On the day of the experiment, the subjects were instructed to lay in the scanner and read the chapter as naturally as possible while remaining alert.

fMRI procedure

Functional images were acquired on a Siemens Verio 3.0T scanner (Siemens, Erlangen, Germany) at the Scientific Imaging & Brain Imaging Center at Carnegie Mellon University, using a T2* sensitive echo planar imaging pulse sequence with repetition time (TR)=2s, echo time=29 ms, flip angle=79°, 36 slices and $3 \times 3 \times 3$mm voxels. Anatomical volumes were acquired with a T1-weighted 3D-MPRAGE pulse sequence.

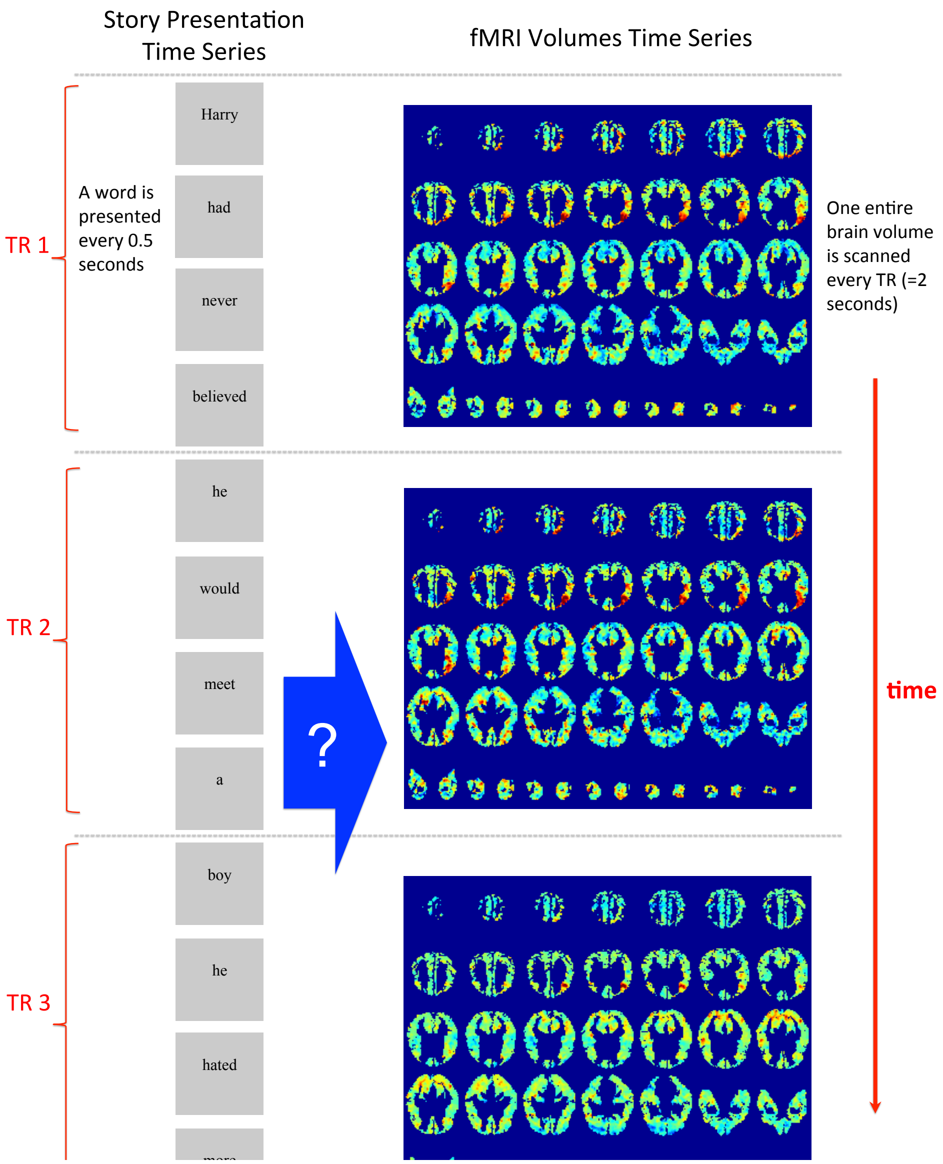

Figure 5. Illustration of our fMRI experimental protocol. Words from a story are presented serially for 0.5 seconds each while recording brain activity with fMRI at a rate of one entire brain image each 2 seconds. Our goal is to model how fMRI neural activity during reading reflects the perceptual and conceptual features of the story. Each fMRI activity volume is shown here in 36 horizontal slices. Going right to left through the slices, then bottom-up, corresponds to looking at slices from the bottom of the brain up. Within each slice, the top of the slice corresponds to the posterior of the brain, and the right side of the slice corresponds to the left side of the brain. The images are on a scale from blue to red where blue indicates negative deviation from baseline and red indicates positive deviations. A TR is the time needed to record one brain volume, and is 2 seconds in our experiment.

Data preprocessing

We used the MATLAB suite SPM8 [Ashburner et al., 2008] to preprocess the data. Each subject's functional data underwent realignment, slice timing correction and co-registration with the subject's anatomical scan, which was segmented into grey and white matter and cerebro-spinal fluid. The subject's scans were normalized to the Montreal Neurological Institute (MNI) space and smoothed with a $6 \times 6 \times 6$mm Gaussian kernel smoother.

Using the Python toolbox PyMVPA [Hanke et al., 2009], we masked the functional data using the segmented anatomical mask, discarding cerebrospinal-fluid voxels. The data was then detrended in MATLAB by running a high-pass filter with a cut-off frequency of 0.005Hz. Visual inspection of the time course of a large number of voxels showed that this threshold was enough to get rid of large block effects and slow trends in the data.

Finally, we selected voxels from each subject, keeping only voxels in 78 cortical Regions Of Interest (ROIs), defined using the AAL brain atlas [Tzourio-Mazoyer et al., 2002], excluding the cerebellum and white matter. We ended up with an average of 29227 voxels per subject. The anatomical union (number of MNI voxel locations for which at least one subject had a voxel) of these 6 subject's brains was a set of 41073 voxel locations.

Appendix B. Representing stories in a feature space

We represented our story features as a multivariate discrete time series. We used one TR as a unit of time. This enables us to have the same time scale for the features and the data time series. We compute the value of a feature at any TR by aggregating the features of the four words that were read during that TR (see table 2).

We extracted the story features at multiple levels of representation. Specifically, we obtained simple perceptual features such as the average word-length in a TR, as well as semantic features of individual words and sentence level features such as syntactic dependency relationship. We also included discourse level features such as the presence of different story characters. The list of all the features we used is provided in tables 1. This table includes the features that were finally used in the model: a few of our features had too few occurrences and we ended up disregarding them. We also include in table 2 as illustration a subset of the feature values for the two segments of the story included in Fig. 2(B).

Visual features

- **Average Word Length:** We compute the average word length in every TR.
- **Word Length Variance:** We compute the variance of word length in every TR.

Semantic features

An approximation of the meaning of a word can be obtained by the pattern of its occurrence with other words over a large text corpus. For example, “apple” is likely to occur with other food items or the verb “eat”, but not so likely to occur with building materials or power tools. These statistics are very large in dimension and therefore we need to resort to some form of dimensionality reduction.

We used NNSE (Non-Negative Sparse Embedding) [Murphy et al., 2012] a which produces low dimensional representations for word meanings that are interpretable and cognitively plausible. The intuition is that, when asked to name the semantic properties of an object, one would list the few salient positive properties (e.g. an apple is a round, usually red, edible object) instead of naming negative properties (e.g. an apple is not a tool), see [Murphy et al., 2012] for more detail. These are learned from massive web corpora from which dependency co-occurrences and document co-occurrence counts are computed. These statistics are then factorized using NNSE.

For every word in our story, we therefore obtain 1000 NNSE features of which we keep the top 100 (these 100 features are picked from the 1000 based on the set of words in the story by choosing the dimensions with the highest average magnitude for these words, whereas the original 1000 were picked by the NNSE model based on the set of all words in the corpora). We sum the features of the four words within each TR.

Syntactic features

Using an automated parser [Nivre et al., 2007] we determined the part of speech of every word in the story and obtained the dependency role of every word from the parse tree of the sentences.

We obtained a set of 28 unique parts of speech and 17 unique dependency relationships, for a total of 45 syntactic binary features that indicate if a given part of speech or a dependency relationship occurred within a TR. We also included an additional feature that records the position of a word in the sentence, i.e. its number starting from the beginning of the sentence. This value is averaged for the four words in a TR.

Discourse features

We made the following annotations manually by going through the story text:

- **Characters:** We resolve all pronouns to the character to whom they refer, and make binary features to signal which of the 10 characters are mentioned.
- **Motions:** We identified a set of motions that occurred frequently in the chapter (e.g. fly, manipulate, collide physically, etc.). Because the actions happen in the course of a sentence, we created two story features for: a punctual feature and a "sticky" feature. The punctual feature represented when the verb of the motion was mentioned, and the sticky feature is on for the duration of the motion (i.e. the sentence). Because we disregarded some of the story features which had few occurrences, we ended up with some motion features that consist only of the sticky feature.
- **Speech:** We indicated the parts of the story that corresponded to direct speech between the characters. We have a punctual feature that indicates the verb that announces which character is speaking (e.g. "said Harry"), and a sticky feature that indicates ongoing direct speech.
- **Emotions:** We identified a set of emotions that were felt by the characters in the chapter (e.g. annoyance, nervousness, pride, etc.). We had punctual features for when the emotion was explicitly mentioned, and sticky features when it was being felt by the characters.
- **Verbs: (non-motion)** We identified a set of actions that occurred frequently in the chapter that were distinct from motion (e.g. hear, know, see, etc.). These typically spanned a shorter time than motions and we only used punctual features to represent them.

Table 1. List of all the textual features.

Semantics	1...100	Syntax	150 Sentence Length
Speech	101 speak - sticky	-parts of speech	151 ,
	102 speak - puntual		152 .
Motion	103 fly - sticky		153 :
	104 manipulate - sticky		154 Coordinating conjunction
	105 move - sticky		155 Cardinal number
	106 collide physically - sticky		156 Determiner
	107 fly - punctual		157 Preposition / sub. conjunction
	108 manipulate - punctual		158 Adjective
	109 move - puntual		159 Modal
Emotion	110 annoyed - puntual		160 Noun, singular or mass
	111 commanding - puntual		161 Noun, plural
	112 dislike - puntual		162 Proper noun, singular
	113 fear - puntual		163 Proper noun, plural
	114 like - punctual		164 Personal pronoun
	115 nervousness - puntual		165 Possessive pronoun
	116 questioning - punctual		166 Adverb
	117 wonder - punctual		167 Particle
	118 annoyed - sticky		168 to
	119 commanding - sticky		169 Interjection
	120 cynical - sticky		170 Verb, base form
	121 dislike - sticky		171 Verb, past tense
	122 fear - sticky		172 Verb, gerung or present part.
	123 mental hurting - sticky		173 Verb, past part.
	124 physical hurting - sticky		174 Verb, non-3rd person sing. present
	125 like - sticky		175 Verb, 3rd person sing. present
	126 nervoussness - sticky		176 Wh-determiner
	127 pleading - sticky		177 Wh-pronoun
	128 praising - sticky		178 Wh-adverb
	129 pride - sticky	-dependency roles	179 Unclassified adverbial
	130 questioning - sticky		180 Modifier or adjective or adverb
	131 relief - sticky		181 Coordination
	132 wonder - sticky		182 Coordination
Verbs	133 be		183 Other dependent (default label)
	134 hear		184 Indirect object
	135 know		185 Modifier of noun
	136 see		186 Object
	137 tell		187 Punctuation
Characters	138 Draco		188 Modifier of preposition
	139 Filch		189 Predicative complement
	140 Harry		190 Parenthetical
	141 Hermione		191 Particle
	142 Mrs. Hooch		192 Root
	143 Mrs. McGonagall		193 Subject
	144 Neville		194 Verb chain
	145 Peeves		195 Modifier of verb
	146 Ron		
	147 Wood		
Visual	148 Average Word Length		
	149 Variance of Word Length		

Table 2. Example of the time course of the different types of story features for two story passages. Stories have to be represented in a feature space that allows for learning the brain response to individual features. The neural response to a novel part of the story can then be predicted as the combination of the responses associated with its features.

	They were half hoping	for a reason to	fight Malfoy, but Professor	McGonagall, who could spot	...	Harry had heard Fred	and George Weasley complain	about the school brooms,	saying that some of
Semantic 1	0	0	0.12	0		0.13	0.11	0	0.01
speak - sticky	0	0	0	0		0	0	0	0
fly - sticky	0	0	0	0		0	0	0	0
manipulate - sticky	0	0	0	0		0	0	0	0
move - sticky	0	0	0	0		0	0	0	0
collide physically - sticky	0	0	0	0		0	0	0	0
hear	0	0	0	0		1	0	0	0
Draco	0	0	1	0		0	0	0	0
Filch	0	0	0	0		0	0	0	0
Harry	1	0	0	0		1	0	0	0
Hermione	0	0	0	0		0	0	0	0
Mrs. Hooch	0	0	0	0		0	0	0	0
Mrs. McGonagall	0	0	0	1		0	0	0	0
Average Word Length	4.5	3	6	5.75		4.25	6	5.25	4
Personal pronoun	1	0	0	0		0	0	0	0
Possessive pronoun	0	0	0	0		0	0	0	0
Object	0	0	1	0		0	1	0	0
Verb chain	1	0	0	1		1	0	0	0

Appendix C. Modeling the time dynamics of the neural activity

We aim to find the mapping between the different types of features we presented above and the neural activity y_v of a voxel v. We want to learn the response of this voxel v to every feature j.

We first assume that each feature j has a signature activity in voxel i that is consistently repeated every time the brain encounters this feature (for the regions that do not encode this feature, we will ideally learn a signature activity equal to 0). Fig. 1(a) shows a hypothetical pattern of activation elicited by the semantic feature j in a given voxel. Due to the TR = 2 seconds we use in our experiment, and the typical latency of the hemodynamic response, we are only interested in the points of the response signature that are sampled 2, 4, 6 and 8 seconds after the onset of feature j (w_1^{vj}, w_2^{vj}, w_3^{vj} and w_4^{vj}). It is important to note that we do not constrain the shape of the learned response signature. We also tried estimating the response with 5 time points (2 to 10 seconds after onset) and 6 time points (2 to 12 seconds). However this manipulation did not significantly change the performance and therefore we use 4 time points for computational and statistical reasons (see Appendix F).

The second assumption is that the signature activity is scaled by the value of feature j at the time the feature is presented. See Fig. 1(b).

Therefore, if we assume that the responses created by successive occurrences of a feature are additive then the activity at time t in voxel v is:

$$y_v(t) = \sum_{k=1}^{4} f_j(t-k) \times w_k^{vj} \tag{1}$$

where $f_j(t)$ is the value of feature j at time t. Another way to think about this is that the activity created by the feature is the convolution of the response signature with the time course of the feature. Above we considered the brain activity to be created by one story feature. Now we include the activities created by all of the features we have defined above, again assuming they are additive. This gives the model:

$$y_v(t) = \sum_{j=1}^{F} \sum_{k=1}^{4} f_j(t-k) \times w_k^{vj} \tag{2}$$

We therefore model the voxel's activity $y_v(t)$ as a linear combination of the values of all the features at times $t-4$ to $t-1$. We know the time courses of the feature values and the voxel's activity, and we need to predict the set of response signatures.

Our approach is similar to Hidden Process Models [Hutchinson et al., 2009] that also use a multiple regression setup. The neural activity there is also assumed to be generated by linearly additive processes and all instantiations of the same process share the same response, but unlike the case of our model, the delay in the onset of the response is variable.

Appendix D. Learning the Response Signatures

In equation 2, we did not consider different subjects, and only considered a hypothetical voxel i. However, in reality, we have S subjects, and $V_T^{(s)}$ voxels for each subject. The regression in equation 2 can therefore be rewritten as:

$$\mathbf{y}_v^{(s)} = \mathbf{F} \times \mathbf{w}_v^{(s)} + \epsilon_v^{(s)} \tag{3}$$

where:

- s is the index of a given subject ($1 \leq s \leq S$)
- n is the number of TRs (or time points)
- $\mathbf{y}_v^{(s)}$ is the $n \times 1$ vector of activity of voxel v of subject s
- $\mathbf{F}$ is the $n \times K$ matrix of time shifted features (every row contains the features of the 4 previous TR, i.e. $K = 4 \times F$)
- $\mathbf{w}_v^{(s)}$ is the $K \times 1$ vector of response signatures in voxel v of subject s
- $\epsilon_v^{(s)} \sim N(0, \sigma_v^2 \mathbf{I}_n)$ is the $n \times 1$ vector of errors (n is the number of TRs) caused by noise in voxel v of subject s (σ_v^2 is the noise variance at voxel v and $\mathbf{I}_n$ is the $n \times n$ identity matrix).

To learn the responses $\mathbf{w}_v^{(s)}$, we solve the following ℓ_2 regularized regression:

$$\min_{\mathbf{w_v}} ||\mathbf{y_v^{(s)}} - \mathbf{F} \times \mathbf{w_v^{(s)}}||_2^2 + \lambda_v^{(s)} ||\mathbf{w_v^{(s)}}||_2^2 \tag{4}$$

independently, for each voxel v and each subject s. This equation has a closed form solution

$$\hat{\mathbf{w}}_v^{(s)} = (\mathbf{F}^\top \mathbf{F} + \lambda_v^{(s)} \mathbf{I}_K)^{-1} \mathbf{F}^\top \mathbf{y_v^{(s)}} \tag{5}$$

where $\mathbf{I}_K$ is the $K \times K$ identity matrix, and we choose the $\lambda_v^{(s)}$ parameter using generalized cross validation [Golub et al., 1979] to estimate the average leave one out cross validation error for each value of $\lambda_v^{(s)}$. Note that at each voxel we are estimating a value for the best regularization parameter independently of the other voxels.

One additional detail is that, for each experimental block, we throw out the first 10 TRs corresponding to the fixation period and the following 1 TR. This 1 TR correspond to the start of the text display and because of the time shift of the features, the feature matrix at that TR has no content (it corresponds to the story features from the 4 following TRs, i.e. the fixation period).

Appendix E. Learned Waveforms

After learning the set of parameters, we look at the four points we learned for a feature j at a voxel v and examine their relative shape. We find that the responses learned are very noisy. However when only looking at the average response for a given feature type at the regions that represent this feature type (we obtain these regions via the classification task explained in detail in the next section), we end up with 4 points that can usually be fitted on a concave waveform that resemble the characteristic shape of the hemodynamic response. We present the average waveforms we learned in Fig. 6. It should be noted that these plots are the averages by feature set, for one of the subjects, of parameters learned across the voxels whose accuracy is in the top 95% percentile, and therefore they are only provided as an illustration.

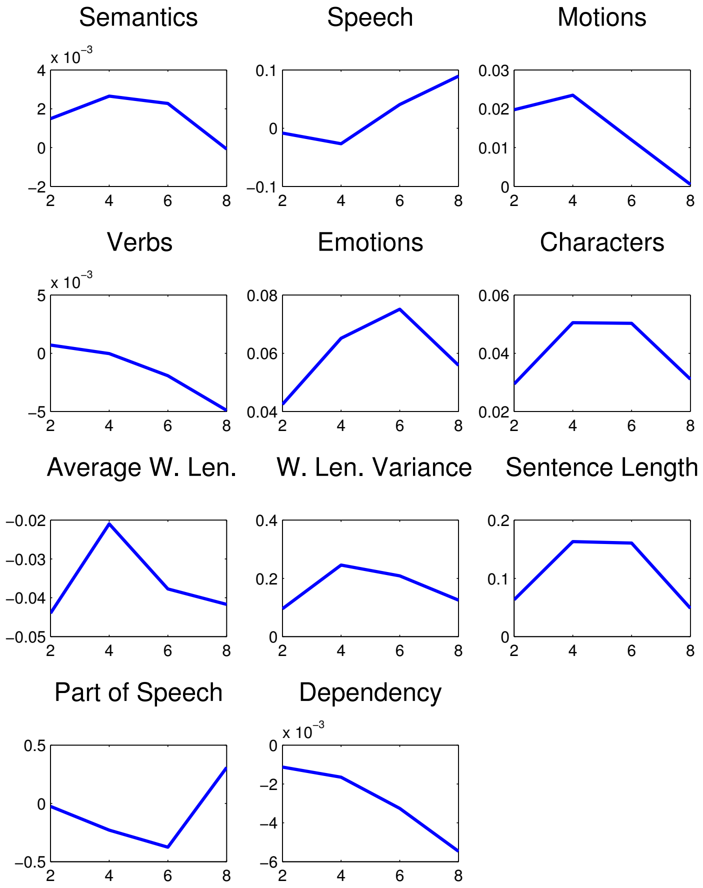

Figure 6. Global averages of the parameters learned for each feature type.

Appendix F. Classification

Cross-Validation Procedure

To learn the response signatures we time-shift the story feature matrix: we make matrix $\mathbf{F}$ in which **every row** t **contains the values of all the features at times** $t-4$**,** $t-3$**,** $t-2$ **and** $t-1$ .We also create an fMRI data matrix containing in **each row t the concatenation of the entire brain images for all subjects, at TR** t.

We introduce here the matrix $\mathbf{W}$, which is the concatenation of all the vectors $\mathbf{w}_v^{(s)}$, i.e.

$$\mathbf{W} = [\mathbf{W^{(1)}}, \mathbf{W^{(2)}} ... \mathbf{W^{(S)}}] \tag{6}$$

where

$$\mathbf{W^{(s)}} = [\mathbf{w}_1^{(s)}, \mathbf{w}_2^{(s)}, ..., \mathbf{w}_{V_T^s}^{(s)}] \tag{7}$$

To test the validity of the learned response signatures, we constructed a binary classifier that decodes which passage of the story is being read from a given fMRI data frame. We start by partitioning the timeline into 10 cross-validation folds. Then for every fold i:

1. Decorrelate the test (fold i) and training data (other 9 folds) by discarding the data corresponding to the 5 TRs before and after fold i.

2. Use the training data to estimate the response signatures of all features in all voxels and all subjects ($\mathbf{W}$), using the method in Appendix D. **It is important to note that the responses are learned independently for each voxel and each subject**. Also note that the penalty parameter for each voxel that is described in Appendix D is chosen using only the training data.

3. Divide the timeline of fold i into non-overlapping time windows, each of length B TRs. Then, for every pair of B TRs segments:

   (a) Take the two test story-frames ($\mathbf{S_1}$ and $\mathbf{S_2}$) and predict the corresponding brain activity using the learned responses $\mathbf{W}$, as shown in Fig. 7.

   (b) Use the two predictions $\mathbf{P_1}$ and $\mathbf{P_2}$ to classify each of the two test data-frames $\mathbf{T_1}$ and $\mathbf{T_2}$ independently: i.e. assign to each data-frame the story-frame with the closest prediction, using a distance function explained in the following subsection.

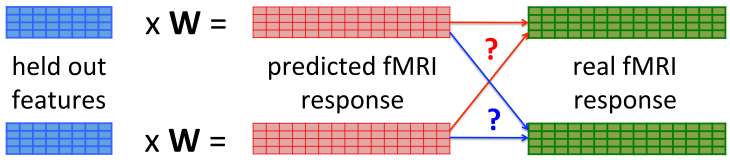

Figure 7. Diagram of the classification task. The task is to assign to each held-out K TRs fMRI segment ($\mathbf{T_1}$ and $\mathbf{T_2}$) the $K \times 2$ seconds portion of the story to which it corresponds (one of the the two dark blue segments). This is done by predicting the activity using the learned weights, then computing the distance between the two predicted responses ($\mathbf{P_1}$ and $\mathbf{P_2}$) and the real segment. The classification of $\mathbf{T_1}$ and $\mathbf{T_2}$ is done independently, i.e. for $\mathbf{T_1}$, the story passage S_1 or S_2 is chosen, and then, in a different test, for $\mathbf{T_2}$, the story passage S_1 or S_2 is chosen.

We average the results of all the cross-validation folds and obtain an overall classification accuracy.

Classification Procedure

Here we describe how the distances between a test segment $\mathbf{T}$ and the two predicted segments $\mathbf{P_1}$ and $\mathbf{P_2}$ that we compare it to are computed (see Fig. 7). We use two methods:

- **Whole-Brain** classification:

  The simplest way to perform classification is to use all the voxels from all the subjects in order to determine the distance between the predicted segments and the true segment. Because we are working with single trial data, concatenating the voxels from different subjects in a row acts as a substitute for multiple repetitions. We compute the Euclidean distance between the two images: $||\mathbf{T}-\mathbf{P_1}||_\mathbf{2}$ and $||\mathbf{T}-\mathbf{P_2}||_\mathbf{2}$.

  Importantly, this test method combine data from multiple subjects without averaging data over subjects in either the learning step (as we saw above) or the classification step. The multi-TR segment that we are classify is actually a multi-TR concatenation of brain images from all subjects, instead of a multi-TR segment of one brain's images. Since every voxel in that data is trained independently, and contributes to the Euclidean distance independently, then this concatenation does not make any assumptions on the subject's alignment.

  As discussed in Appendix G and the main text, when using the data that has been smoothed in preprocessing, the classification accuracy is lower than when using un-smoothed data. Therefore we boost the accuracy when using smoothed data by voxel selection. This is done in the following way: at every cross-validation fold, we use the training data in order to find the best subset of voxels to use. This is done via a nested cross-validation step on the training data what determines which voxels have the best accuracy and how many of the top voxels to use to obtain the best combined accuracy. These voxels are then used to classify the untouched test data: we compute the Euclidean distance using only the columns that correspond to these voxels.

- **Concatenated Searchlight** classification:

  Whole-Brain accuracies do not tell us about which parts of the brain are contributing to the classification accuracy. In order to assess this, we perform the classification "locally", looking in one region of the brain at a time. Regions are defined as $k \times k \times k$-voxel cubes centered around one MNI voxel location, k being an odd integer. This method is similar to the Searchlight approach commonly used in neuroimaging [Kriegeskorte et al., 2006], however we expand it to include data from multiple subjects:

  - We pick a cube size k: for example, $k = 5$ gives a $5 \times 5 \times 5$ voxels cube (to look at one voxel at a time we take a $1 \times 1 \times 1$ voxel cube)
  - For every voxel location (x_i, y_i, z_i), we select the set of voxels whose coordinates fall in the $k \times k \times k$ voxels cube centered around that location. This can be done for each subject independently, in the case where we are interested to look for regions with high accuracy on a single subject basis. It can also be done by selecting the union of voxels from all subjects that fall in this cube. We call the set of voxels selected at this step $\mathbf{V}_i$.

    Because we are working with single trial data, concatenating the corresponding voxels from different subjects in a row acts as a substitute for multiple repetitions. Additionally, since the alignment of the subjects to the same anatomical space is not perfect, taking a $k \times k \times k$ voxel cube with $k > 1$, allows us to circumvent small variations in the anatomical configuration of the subjects brains.
  - For each of these sets $\mathbf{V}_i$ of voxels, we compute the Euclidean distances:

    $||\mathbf{T}(\text{all rows, voxels in } \mathbf{V}_i) - \mathbf{P_1}(\text{all rows, voxels in } \mathbf{V}_i)||_2$ and

    $||\mathbf{T}(\text{all rows, voxels in } \mathbf{V}_i) - \mathbf{P_2}(\text{all rows, voxels in } \mathbf{V}_i)||_2$

Note: we are performing this computation at every voxel, so we are actually performing N_v classifications, where N_v is the total number of voxels.

Significance Testing

- **Whole-Brain Classification Accuracy**

  To show that Whole-Brain classification accuracy is significantly higher than chance accuracy, which is 50% in this balanced binary classification task, we compute an empirical null distribution. The null distribution that story features cannot predict neural activity is approximated empirically. A common approach to estimate the null distribution is by running a permutation test: the order of the features is permuted before classification and the procedure is repeated a large number of time. However, the different samples (different TRs) of our experiment are not identically and independently distributed (IID) given that the data is from a time series. The time series of data and of features varies smoothly and therefore the classifier might detect dependencies between them when there is none, because they happen to vary similarly in this finite sample. The commonly used permutation test will not contain such dependencies and therefore will not correct for them, therefore leading to an optimistically biased answer. To solve this problem we use a solution inspired by [Chwialkowski and Gretton, 2014]: we shift the feature time series by N TRs such that $a < N < b$ and compute the classification accuracy. For a and b large enough (we use $a = 500$ and $b = 750$), there will be no real relationship between the time series of data and the time series of features, however the time smoothness will be conserved, leading to better estimates of the variance of chance classification accuracy, which guarantees less false positives.

- **Identifying Brain Regions Correlated with Different Feature Types:**

  To find out where in the brain each type of story feature is useful, we followed a similar training approach as previously, except that (1) only one type of feature (Word Length, Story Characters etc...) was used at a time and (2) we used a concatenated Searchlight procedure at test time with $k = 5$ and using data from all subjects. Precisely, for every voxel location i, we took the cube of $5 \times 5 \times 5$ voxel coordinates centered around that location. The union of voxels from all subjects that have coordinates included in this cube were selected. Therefore, for every location, we performed the classification of 2 segments of size $20 \times |\mathbf{V}_i|$.

  For every one of these combination of type of story feature/subset of data, we obtain a local classification accuracy. We measure significance by computing an empirical null distribution in the same way as for the whole-brain accuracy, then correcting for multiple comparisons using the Benjamini-Hochberg-Yekutieli False Discovery Rate (FDR) [Benjamini and Yekutieli, 2001]. This procedure controls the FDR at level q under arbitrary dependence and therefore we did not need to make independence assumptions about the accuracies of different voxels. The procedure is, for N comparisons:

  - Sort the N p-values.
  - Find the largest j such that
    $$p_{(j)} \leq \frac{j}{N} \times \frac{q}{(\sum_{i=1}^{N} \frac{1}{i})}.$$
  - Reject the null hypothesis for the j comparisons with the smallest p-values.

Testing the number of time-points

After computing the accuracy and the chance distribution at every voxel and for every feature, we repeat the entire experiment with more estimated time points per response signature: 5 and 6, corresponding respectively to points 2 to 10s and 2 to 12s after feature presentation. While the obtained patterns of representation vary slightly, we do not find any region in which there is a significant improvement for using either type of window. Since the performance is not different, we chose to use 4 because of statistical concerns: we have a training set of about 1100 points and 195 features, it is more advisable to limit the amount of covariates when estimating the model.

Appendix G. Additional results

We present here the 3D map we obtain for syntactic features exclusively, divided into the contribution from our three types of syntactic features: sentence length, part of speech and dependency roles.

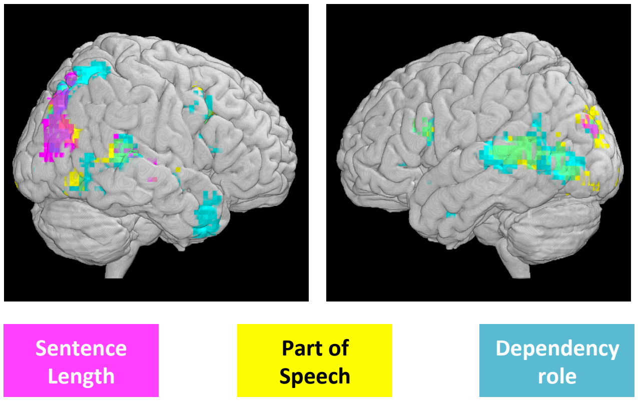

Figure 8. Results obtained by our generative model for different syntax features, showing where sentence length, part of speech, and dependency roles are encoded by neural activity. Each voxel location represents the classification when using a cube of $5 \times 5 \times 5$ voxel coordinates, centered at that location, such that the union of voxels from all subjects whose coordinates are in that cube are used. Voxel locations are colored according to the feature set that can be used to yield significantly higher than chance accuracy.

We have also ran the entire experiment with the same setup, using however the data without spatial smoothing. The results vary to a considerable degree in the boundaries of each region, while the main location of each feature representation stays the same. Figures 9 and 10 show the resulting maps.

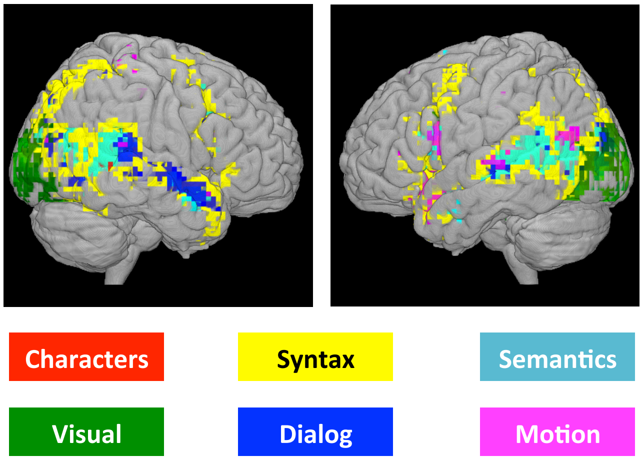

Figure 9. Same as figure 4 with non-smoothed data (at FDR $\alpha = 0.01$).

Our results do not only depend on processing methods, but they also require the significance thresholding of different classification tasks which might not be of equal difficulty. For instance, different features might lead to high or low classification because of the statistical properties of the features and not the

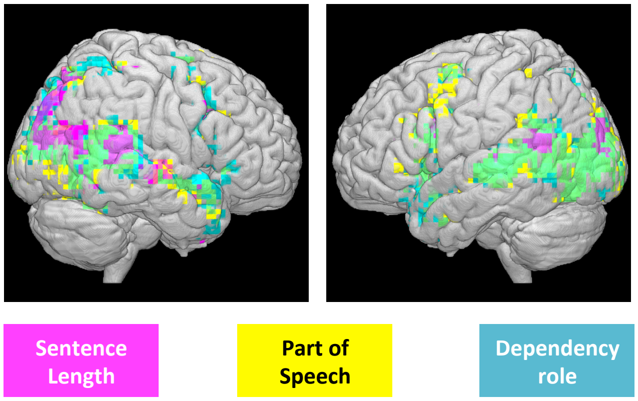

Figure 10. Same as figure 8 with non-smoothed data.

way they are represented in the brain. We present below the comparison of the whole brain classification when different types of features are used. We compare these accuracies with the entropy of each feature set. We want to see if the difference in classification accuracy is due to differences in the entropy of each feature: it is harder to learn a model with features that change rarely in a story (low entropy), than it is to learn a model with features that occur very frequently. In our feature creation phase, we did explicitly exclude features with low entropy (for example, the location of scenes didn't vary much and we didn't include it). However, the features we did keep still vary in their frequency and we wanted to compare their entropies to their accuracies.

For each feature set we compute the entropy of each feature, and then use the maximum entropy. The results are shown in the first row of tables 3 and 4. In the following rows, we show classification accuracy by feature set. For the smoothed data, the accuracy was initially low and was boosted by voxel selection as explained in Appendix F.

	NNSE	Average WL	Variance WL	Sentence Length
entropy	1.84	4.01	5.22	5.46
accuracy (smoothed)	0.58	0.69	0.57	0.53
boosted accuracy (smoothed)	0.63	0.87	0.80	0.62
accuracy (unsmoothed)	0.75	0.71	0.71	0.67

Table 3. Non-binary features.

	speak	move	emotions	verbs	characters	POS	dependency
entropy	0.69	0.50	0.25	0.28	0.28	0.62	0.78
accuracy (smoothed)	0.55	0.51	0.50	0.51	0.56	0.61	0.62
boosted accuracy (smoothed)	0.66	0.61	0.50	0.65	0.52	0.71	0.71
accuracy (unsmoothed)	0.69	0.61	0.48	0.65	0.56	0.84	0.83

Table 4. Binary features.

There seems to be a modest relationship between the entropy of the features and how accurate classification is, in which feature sets with higher entropy lead to a higher accuracy. There might be other factors also affecting how easy the classification with different feature sets are. To avoid comparing the results of classification tasks that vary in difficulty, and as a way of leveling the playing field, we plot

in Fig. 11 the top 1000 voxels when using each of the feature sets. The voxels that are colored do not therefore necessarily have a higher than chance classification accuracy.

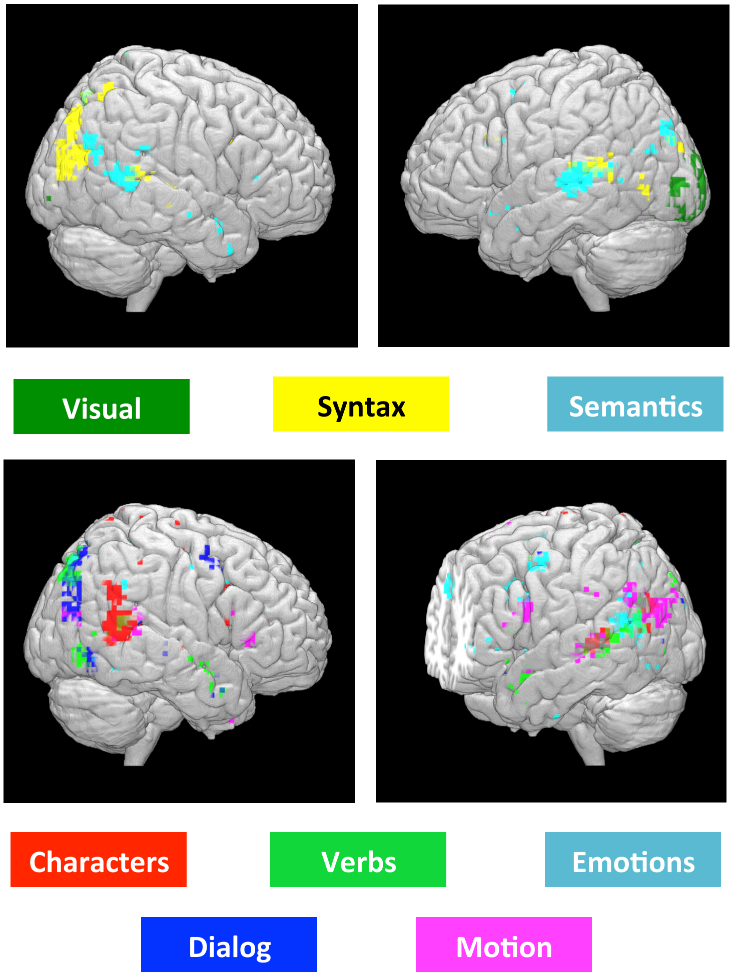

Figure 11. Top 1000 voxels for each feature type (smoothed data). Instead of picking the significantly higher than chance voxels, we chose to color the 1000 voxels with the highest (normalized) accuracy for each feature type. The accuracies were normalized using the empirical null distribution as explained in Appendix F. In the lower, right figure, the brain is sliced to reveal in the medial frontal cortex a cluster of voxels that in which emotions lead to relatively high accuracy.

Appendix H. Combining Subjects Spatially

Our concatenated Searchlight is not equivalent to spatial or cross-participant smoothing because, again, the voxels associated with each subject are treated independently. The only requirement is that the subjects are all normalized to the MNI space; we do not co-register the subjects and we learn the response of every voxel independently.

Assume we are interested in an area A that is distributed around a certain mean location (x, y, z) in all subjects. Then despite the subjects' anatomical variability and given an adequate model and an appropriate cube-size, the cube centered at (x, y, z) will contain in it the voxels from area A of all subjects. Running the classification at this cube should then hypothetically yield the best accuracy. This would be possible because, inside the cube, the voxels from all subjects are concatenated and they contribute independently to the Euclidean distance we compute in classification. The voxels' precise alignment is irrelevant at this step, it only matters that they are all taken into consideration. Therefore, this method identifies regions of a given size (in this case $15\text{mm} \times 15\text{mm} \times 15\text{mm}$) in which the subjects are processing the same information. It avoids the problem usually encountered in averaging multiple subjects, which is that the only regions that are identified are the regions in which the subjects highly overlap. This problem is widely debated in the literature [Fedorenko et al., 2010].

Furthermore, despite the linearity of the model, this approach does not yield the same results as spatially smoothing the data in the cubes, because we have a multivariate input (the different story features) and while nearby voxels might be processing the same type of information (e.g. story characters), they are hypothetically coding different instances of this information (e.g. different story characters) with different patterns of activity for each instance.

References

[Ashburner et al., 2008] Ashburner, J., Chen, C., Flandin, G., Henson, R., Kiebel, S., Kilner, J., Litvak, V., Moran, R., Penny, W., Stephan, K., et al. (2008). SPM8 manual. *Functional Imaging Laboratory, Institute of Neurology.*

[Benjamini and Yekutieli, 2001] Benjamini, Y. and Yekutieli, D. (2001). The control of the false discovery rate in multiple testing under dependency. *Annals of statistics*, pages 1165–1188.

[Brainard, 1997] Brainard, D. (1997). The psychophysics toolbox. *Spatial vision*, 10(4):433–436.

[Buchweitz et al., 2009] Buchweitz, A., Mason, R., Tomitch, L., and Just, M. (2009). Brain activation for reading and listening comprehension: An fMRI study of modality effects and individual differences in language comprehension. *Psychology & Neuroscience*, 2(2):111–123.

[Chwialkowski and Gretton, 2014] Chwialkowski, K. and Gretton, A. (2014). A kernel independence test for random processes. *arXiv preprint arXiv:1402.4501.*

[Fedorenko et al., 2010] Fedorenko, E., Hsieh, P.-J., Nieto-Castanon, A., Whitfield-Gabrieli, S., and Kanwisher, N. (2010). New method for fMRI investigations of language: Defining ROIs functionally in individual subjects. *Journal of Neurophysiology*, 104(2):1177–1194.

[Golub et al., 1979] Golub, G. H., Heath, M., and Wahba, G. (1979). Generalized cross-validation as a method for choosing a good ridge parameter. *Technometrics*, 21(2):215–223.

[Hanke et al., 2009] Hanke, M., Halchenko, Y., Sederberg, P., Hanson, S., Haxby, J., and Pollmann, S. (2009). Pymvpa: A Python toolbox for multivariate pattern analysis of fMRI data. *Neuroinformatics*, 7(1):37–53.

[Hutchinson et al., 2009] Hutchinson, R., Niculescu, R., Keller, T., Rustandi, I., Mitchell, T., et al. (2009). Modeling fMRI data generated by overlapping cognitive processes with unknown onsets using Hidden Process Models. *NeuroImage*, 46(1):87–104.

[Kleiner et al., 2007] Kleiner, M., Brainard, D., Pelli, D., Ingling, A., Murray, R., and Broussard, C. (2007). What's new in Psychtoolbox-3. *Perception*, 36(14):1–1.

[Kriegeskorte et al., 2006] Kriegeskorte, N., Goebel, R., and Bandettini, P. (2006). Information-based functional brain mapping. *Proceedings of the National Academy of Sciences of the United States of America*, 103(10):3863–3868.

[Murphy et al., 2012] Murphy, B., Talukdar, P., and Mitchell, T. (2012). Learning effective and interpretable semantic models using Non-Negative Sparse Embedding. In *International Conference on Computational Linguistics (COLING 2012), Mumbai, India.*

[Nivre et al., 2007] Nivre, J., Hall, J., Nilsson, J., Chanev, A., Eryigit, G., Kubler, S., Marinov, S., and Marsi, E. (2007). Maltparser: A language-independent system for data-driven dependency parsing. *Natural Language Engineering*, 13(2):95.

[Pelli, 1997] Pelli, D. (1997). The VideoToolbox software for visual psychophysics: Transforming numbers into movies. *Spatial vision*, 10(4):437–442.

[Potter, 2006] Potter, B. (2006). *The Tale of Peter Rabbit.* ABDO.

[Rowling, 2012] Rowling, J. (2012). *Harry Potter and the Sorcerer's Stone.* Harry Potter US. Pottermore Limited.

[Tzourio-Mazoyer et al., 2002] Tzourio-Mazoyer, N., Landeau, B., Papathanassiou, D., Crivello, F., Etard, O., Delcroix, N., Mazoyer, B., Joliot, M., et al. (2002). Automated anatomical labeling of activations in SPM using a macroscopic anatomical parcellation of the MNI MRI single-subject brain. *Neuroimage*, 15(1):273–289.
